# Resolution Matters: Correlating Quantitative Proteomics and Nanoscale‐Precision Microscopy for Reconstructing Synapse Identity

**DOI:** 10.1002/pmic.201800139

**Published:** 2018-07-30

**Authors:** Andras Gabor Miklosi, Giorgia Del Favero, Doris Marko, Tibor Harkany, Gert Lubec

**Affiliations:** ^1^ Department of Molecular Neurosciences Center for Brain Research Medical University of Vienna Vienna A‐1090, Austria; ^2^ Department of Food Chemistry and Toxicology Faculty of Chemistry University of Vienna Vienna A‐1090 Austria; ^3^ Department of Neuroscience Karolinska Institutet SE‐17177 Stockholm Sweden; ^4^ Neuroproteomics Laboratory Paracelsus Medical University A‐5020 Salzburg Austria

**Keywords:** dSTORM, immunohistochemistry, spatial proteomics, synapse

## Abstract

For more than a century, the precision at which any protein (or RNA) could be localized in living cells depends on the spatial resolution of microscopy. Light microscopy, even recently benchmarked laser‐scanning microscopy, is inherently liable to the diffraction limit of visible light. Electron microscopy that had existed as the only alternative for decades is, in turn, of low throughput and sensitive to processing artefacts. Therefore, researchers have looked for alternative technologies particularly with ever‐growing interest in resolving structural underpinnings of cellular heterogeneity in the human body. Computational (“in silico”) predictions provided only partial solutions given the incompleteness of existing databases and erroneous assumptions on evolutionarily conserved sequence homology across species. A breakthrough that facilitates subcellular protein localization came with the introduction of “super‐resolution” microscopy, which yields 20–60 nm resolution by overcoming diffraction‐limited technologies. The ensuing combination of “super‐resolution” microscopy with unbiased proteomics continues to produce never‐before‐seen gains by quantitatively addressing the distribution, interaction, turnover, and secretion of proteins in living cells. Here, we illustrate the power of this combined work flow by the example of transmembrane receptor localization at the neuronal synapse. We also discuss how dynamic analysis allows for inferences be made for cellular physiology and pathobiology.

Subcellular localization of proteins provides pivotal information for the investigation and modelling of cellular processes and pathways controlled by protein–protein interactions. Despite decades of investment, most proteins have been inadequately or incompletely localized,^1^ a notion that particularly pertains to subcellular protein distribution in various tissues of the mammalian body.

Proteins identified within large proteomes are commonly assigned to presumed subcellular positions by high‐throughput prediction algorithms exploiting sequence homology and functional classifiers.^2^, ^3^, ^4^, ^5^ Despite inherent ambiguities of “in silico predictions,” they carry significant appeal because of the brevity of analysis time. Unfortunately, experimental pipelines to verify physicochemical assumptions at large scale are rarely built with candidate analysis in many laboratories providing incongruent data quality and conclusions.

In experimental cell biology, antibody‐based immunohistochemical methods became benchmarks of target discovery. For protein localization in tissues that contain diverse cell types, marker proteins are used to “landmark” specific cellular compartments, an approach compatible with multicolor immunofluorescence light microscopy. A quantum leap in the precision of protein localization by light microscopy came with the introduction of confocal microscopy that can maximally separate excitation wavelengths by use of wavelength‐tuned lasers, as well as exclude out‐of‐focus light by applying micrometer‐scale apertures (“pinholes”), which act as low‐pass filters for the light beam. Even with the most advanced systems, light microscopy is diffraction limited: it cannot resolve fine differences in compartmentalization for subwavelength structures ≲250 nm.

Electron microscopy is the alternative localization approach that first came to prominence. When combined with the use of antibodies (termed immunoelectron microscopy), subcellular assignments can be called at ≈5–10 nm precision with the physical size of antibody molecules defining its boundary of stochasticity. However, immunoelectron microscopy is known to be sensitive to processes of tissue preservation, antigen accessibility (e.g., glutaraldehyde‐containing fixatives), antibody impurities, embedding, washing, and contrasting conditions.^6^, ^7^, ^8^, ^9^, ^10^ Given the multistep procedures required, immunoelectron microscopy is of low throughput and demands specialist knowledge to produce reliable results.

Therefore, methods that preserve the benefits of both light and electron microscopy, namely versatility, speed of tissue processing, and quasi‐equal resolution in multiple colors are sought after when designing pipelines for protein profiling. Retaining considerable throughput grew significantly in importance recently given the increasing number of tissue‐specific proteomes in many mammalian species.^1^, ^11^, ^12^ Likewise, maximizing resolution remains a primary concern when i) molecularly heterogeneous cells are situated proximal to one another, ii) analyzing morphologically specialized cell types, often at considerable temporal dynamics (e.g., cell migration and growth, (dis‐)assembly of protein aggregates) or iii) studying molecular determinants of specialized conduits of intercellular communication (e.g., immunological or neuronal synapses).^13^, ^14^, ^15^, ^16^

To satisfy the above objectives, “super‐resolution” microscopy has recently been developed to aid the subcellular localization of proteins by overcoming the resolution cut‐off imposed by the diffraction limit of light.^17^, ^18^, ^19^, ^20^, ^21^ Among the “super‐resolution” microscopy platforms available today, structured illumination microscopy (SIM),^17^ stimulated emission depletion (STED),^22^ and direct stochastic optical reconstruction microscopy (dSTORM)^23^ are particularly well liked because these techniques feature optical resolution in the range of 10–100 nm, can be used on various tissue types (from cell cultures to pathology specimens), and are available for multicolor processing. Inflation microscopy is a recently developed alternative that artificially increases tissue surfaces to separate closely sitting targets for detection by diffraction‐limited microscopy systems. True intermolecular distances are then calculated by factorial arithmetics.^24^, ^25^

Here, we focus on dSTORM by highlighting its experimental prowess when combined with global proteomics. As such, multicolor dSTORM is particularly amenable for subcellular *co‐*localization studies since it aids single‐molecule detection upon high‐density multichannel labelling in large fields of view. Thereby, high‐resolution dSTORM^14^, ^26^ is well suited to map unknown proteins onto structural “landmarks” at considerable versatility and pace, which is compatible with recent developments in large array platforms, including single cell and/or spatial proteomics and transcriptomics.

dSTORM is an extension of photo‐activation localization microscopy (PALM) for the single‐molecule localization of fluorescent proteins.^27^ While PALM relies on genetically engineered chimeras of protein targets and bacterial fluorescent protein tags, dSTORM instead employs conventional fluorescently‐labelled antibodies which can be combined to cover the entire light spectrum. The principal requirement for dSTORM imaging is the establishment of a chemical environment (e.g., by thiol‐reducing agents), which captures and prolongs the existence of a non‐photon‐emitting triplet (or “off”) state of the fluorophores (including Atto, AlexaFluor, and carbocyanines) and facilitates a transition state (intersystem crossing) initiated by laser excitation. Through subsequent oxidative reactions triggered by molecular oxygen in aqueous buffers, powerful energy transfer by laser excitation then allows for a subset of fluorescent dye molecules to recover their photon‐emitting (or “on”) state, which can be captured by recording at high frame‐rates. Multiplying the number of “on/off” cycles distinguishes subsets of individual fluorophores whose positions localize target molecules precisely and reproducibly. Thereby, and in addition to visualization at high density and in multicolor mode, dSTORM was successfully used to reveal novel fine subcellular details including protein clustering and protein–protein interactions (e.g., for receptors and their signal effectors).^28^, ^29^

Here, we exemplify the usefulness of dSTORM in neurobiology with a particular focus on synaptic proteins that are indispensable for intercellular communication in the nervous system. Resolving protein distribution at specialized pre‐ or postsynaptic sites that form the junction of intercellular information transfer between connected nerve cells (that is, the “synapse”) is a central effort in neurobiology to decipher physiological processes, as well as to implicate specific signal transduction cascades in the pathogenesis of neuropsychiatric and neurological diseases. Notably, a myriad of proteins is selectively partitioned into either the presynaptic terminal or the subsynaptic dendrite to specify steps of chemical (or electrical) neurotransmission. Routinely, subcellular fractionation by sequential ultracentrifugation is the standard to separate and enrich cellular organelles. For synaptic proteins, the separation of synaptosomes (i.e., isolated nerve terminals containing intact pre‐ and postsynaptic compartments; Figure 1A,B) is traditionally used to explore protein constituents and determine their physicochemical interactions in signal transduction cascades. The high yield of synaptosomes makes them favorable to test molecular determinants of synaptic neurotransmission, ranging from single proteins to protein networks (including multiprotein complexes) and the dynamic recruitment of protein interactomes (“signalosomes”) when combined with pharmacological probing. Key shortcomings of synaptosome preparations are that biochemistry‐guided proteomics alone lacks precision to reach the state of pure pre‐ or postsynaptic compartments,^30^ the lack of spatial information on a synapse's location in the brain, and that many proteins (e.g., the D1 dopamine receptor^31^) might serve different functions when recruited to pre‐ or postsynaptic sites.

**Figure 1:**
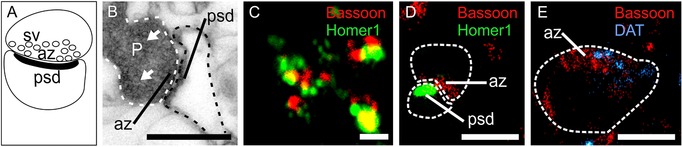
From structural principles to single‐molecule localization. A) Schematic drawing of an isolated synaptosome retaining an intact synapse with its major components: neurotransmitter‐laden synaptic vesicles (*sv*), the presynaptic active zone (*az*), and postsynaptic density (*psd*). B) Representative electron micrograph of an isolated synaptosome showing preserved synaptic architecture with ubiquitous structural compartments labelled. (P, presynaptic terminal). Solid arrowheads point to synaptic vesicles. White and black dashed lines encircle the pre‐ and postsynaptic compartments, respectively. Figure panel was adapted with permission.^29^ Copyright 2017, Springer Nature. Representative images showing differences in resolving the localization of pre‐ and postsynaptic structures with synaptic markers serving as “landmarks” (in red: Bassoon, a presynaptic active zone marker; in green: Homer1, a postsynaptic density marker) by C) confocal microscopy and D) dSTORM. Images correspond to resolution limits described by Miklosi et al. and others.^26^, ^29^, ^35^ E) Localization of DAT (blue) clusters at super‐resolution within the presynaptic active zone (Bassoon, red) shows single molecules and their clusters intracellularly and on the membrane surface. Boundaries of the pre‐ and postsynaptic compartments were delineated in (D,E) (dashed lines). Scale bars = 500 nm (B,C), 200 nm (D,E).

Henceforth, combining synapse proteomics with dSTORM imaging in fixed (and live) cells is amenable to develop benchmarked discovery pipelines encompassing i) protein localization at specialized subcellular sites, ii) quantitation of protein content in physiological and disease settings, iii) resolving protein localization at pre‐ and postsynaptic sites (including dynamic single‐molecule tracking of protein turnover and compartmentalization), iv) localization of synapse contingents with specific features in the brain, v) causally integrating specific proteins in synaptic neurotransmission within diverse neuronal networks, and vi) interrogating transient interactomes at high precision, resolution, and throughput. Notably, dSTORM is a valid method not only as a secondary verification tool (that is, quality control) but also as the choice of method to define subcellular protein organization when functional segregation exists at subcellular domains at the nanometer scale. In neurons, this is best illustrated by the immediacy of synaptic, perisynaptic, and nonsynaptic membrane partitions that host vastly different protein machineries, for example, vesicle fusion, Ca^2+^ regulation, and receptor‐mediated signal transduction.

With the present rate of technical developments, mass‐spectrometry‐assisted proteomics can identify >8000 synaptic proteins (including >2000 intra‐ or transmembrane species) in synaptosomal fractions. Ensuing knowledge on protein localization, spurred by the recent completion of the Human Protein Atlas, heralds a new era for “neuroproteomics.” Since the Human Protein Atlas alone has produced antibodies for >20 000 human proteins and already generated refined maps for subcellular and pathological protein localization,^32^, ^33^ the next challenge will be to device versatile technical platforms to precisely and simultaneously localize these proteins and generate their overlays on structural landmarks at nanoscale precision in tissues^26^ and cultures cells.^29^ dSTORM, when coupled to either repeated bleaching cycles for reprobing samples or to spectrally unmixing a kaleidoscope of colors carried by hundreds of directly‐conjugated primary antibodies applied simultaneously, could fill a critical void to aid next‐generation discoveries. Notwithstanding, further development on quantification strategies in specific cell types, single cells and brain areas, and extending this method to the subcellular (or “single molecule”) localization of RNAs^34^ are expected to produce a census of cellular constituents at unprecedented resolution and reliability.

## Conflict of Interest

The authors declare no conflict of interest.
